# Mosquito breeding water parameters are important determinants for *Microsporidia MB* in the aquatic stages of *Anopheles* species

**DOI:** 10.1186/s13071-024-06596-9

**Published:** 2024-12-18

**Authors:** Esinam A. Akorli, Nana Efua Andoh, Richardson K. Egyirifa, Christopher Dorcoo, Sampson Otoo, Seraphim N. A. Tetteh, Reuben Mwimson Pul, Derrick B. Sackitey, Stephen K. D. Oware, Samuel K. Dadzie, Jewelna Akorli

**Affiliations:** 1 Department of Parasitology, Noguchi Memorial Institute for Medical Research, University of Ghana, Legon, P.O. Box LG 581, Accra, Ghana; 2 Department of Pathology, University of Cambridge, 10 Tennis Ct Rd, Cambridge, CB2 1QP UK

**Keywords:** *Microsporidia MB*, *Anopheles* mosquitoes, Mosquito breeding site, Physicochemical parameters

## Abstract

**Background:**

*Microsporidia MB* disrupts *Plasmodium* development in *Anopheles* mosquitoes, making it a possible biocontrol tool for malaria. As a tool for vector/disease control, its ecological distribution and the factors that determine their occurrence must be defined. We investigated the frequency of *Microsporidia MB* in *Anopheles* mosquitoes across selected sites in northern and southern Ghana, as well as the physicochemical parameters of mosquito breeding water that are associated with the occurrence of the fungus, by fitting regression models.

**Methods:**

A non-column extraction method was used to extract DNA from the abdomens of 4255 adult *Anopheles* mosquitoes that emerged from larvae and pupae collected between August and October of 2021 and 2022. Detection of *Microsporidia MB* was achieved using quantitative PCR (qPCR), while mosquito species were molecularly identified using short interspersed nuclear elements (SINE), restriction fragment length polymorphism (RFLP) methods, and the ANOSPP algorithm.

**Results:**

Overall *Microsporidia MB* distribution was 2.2% (92/4255). Male mosquitoes exhibited a higher frequency of infections and had a predicted probability of infection that was 85% higher than that of females. Sites in Ghana's Savannah zone had the highest *Microsporidia MB* distribution (68.5%). Biochemical oxygen demand in mosquito breeding water was estimated to be positively associated with and significantly predicts *Microsporidia MB* in mosquitoes with an accuracy of 94%. Increasing ammonium ion concentrations reduced the chances of finding *Microsporidia MB*-positive mosquitoes. According to our data, all *Anopheles* mosquitoes, including minor species such as *An. squamosus*, *An. pretoriensis* and *An. rufipes*, had equal probability of *Microsporidia MB* infection.

**Conclusions:**

These results provide preliminary information on micro-ecological factors that potentially support the sustainability of *Microsporidia MB* infection in mosquitoes during their aquatic life stages. It will be important, therefore, to explore the impact of strategies for larval source management on these factors to ensure that the symbiont's persistence during the host's aquatic stages may not be adversely affected should it be used as an integrated approach for mosquito/disease control.

**Graphical abstract:**

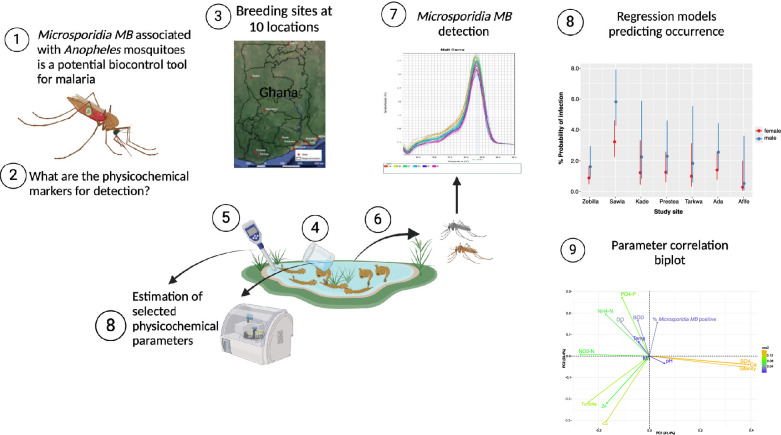

**Supplementary Information:**

The online version contains supplementary material available at 10.1186/s13071-024-06596-9.

## Background

Novel malaria control measures using mosquito symbionts that target the transmission of the *Plasmodium* parasite in malaria vectors are being explored. Candidate anti-parasitic micro-organisms for disease control are required to be generationally transferrable and environmentally restricted to the target hosts and present little or no fitness costs to the vector host, among other characteristics [1–3]. *Microsporidia MB*, a fungus, has been identified to naturally infect major malaria anopheline vectors in sub-Saharan Africa [4–6] and is associated with reduced *Plasmodium* oocysts in infected *Anopheles arabiensis* [5]. Although *Microsporidia MB* has not yet been tested for its effects on *Plasmodium* in *Anopheles gambiae*, this fungus has been reported to be avirulent in this *Anopheles* species and has transmission characteristics [7] similar to those observed in *An. arabiensis* [4]. This makes *Microsporidia MB* a plausible candidate for malaria transmission-blocking in anopheline mosquitoes. Fundamentally, it appears to satisfy most, if not all of the preferred characteristics of an ideal micro-organism for biocontrol of disease.

Larval source management, monitoring programs and an understanding of the ecological determinants of conducive environments for mosquito sustainment remain integral parts of mosquito and disease control. Ultimately, removing larval breeding sites, particularly those created by human activities such as urban farming, would reduce the incidence of mosquitoes and their associated diseases [8–10]. Due to the global rise in temperatures, the increasing incidence of rainfall events resulting from climate change [11, 12] and changes in landscapes due to urbanization [13–16], larval sites continue to emerge in new environments, and mosquitoes are adapting to new niches [17, 18]. Regardless of these changes, larval source mapping and larviciding significantly contributed to disease control [19–22]. Introducing paratransgenic micro-organisms to mosquitoes at larval breeding sites has also been proposed as a potential management strategy [23]. The underlying rationale is that mosquito larvae obtain a substantial part of their gut microbiota from the water in which they breed and can transfer these micro-organisms from one developmental stage to another [24]. In addition, the physicochemical properties of the mosquito larval breeding water impact the microbial diversity at breeding sites, mosquito larval abundance and body size [25–29].

The ecological distribution of *Microsporidia MB* is still not clearly understood. In Kenya, where this fungus was first described in *An. arabiensis*, frequencies were observed to peak some weeks after a rainfall event [5]. The authors of this study suggested that *Microsporidia MB* transmission between mosquitoes was influenced by environmental factors [5]. Other microsporidian species have been particularly observed to be more prevalent at sites where irrigated rice fields are present [30], and *Microsporidia MB* has also been reported near irrigation sites [5]. Whether this is mainly because of the permanent availability of breeding sites, or there are ecological factors that influence its presence, remains undefined. One potential route for using *Microsporidia MB* is by spore dispersal into the environment where mosquitoes would be infected by ingestion [3]; this strategy necessitates a clear understanding of the environmental requirements to explore such opportunities for control. Thus, in this study, we investigated the frequency and distribution of *Microsporidia MB* among *Anopheles* mosquitoes from Ghana, as well as the association of these with selected physicochemical properties in *Anopheles* larval breeding sites. We used regression models to identify those factors that significantly influence the predictability of *Microsporidia* *MB* infection in mosquitoes.

## Methods

### Study areas

**Fig. 1:**
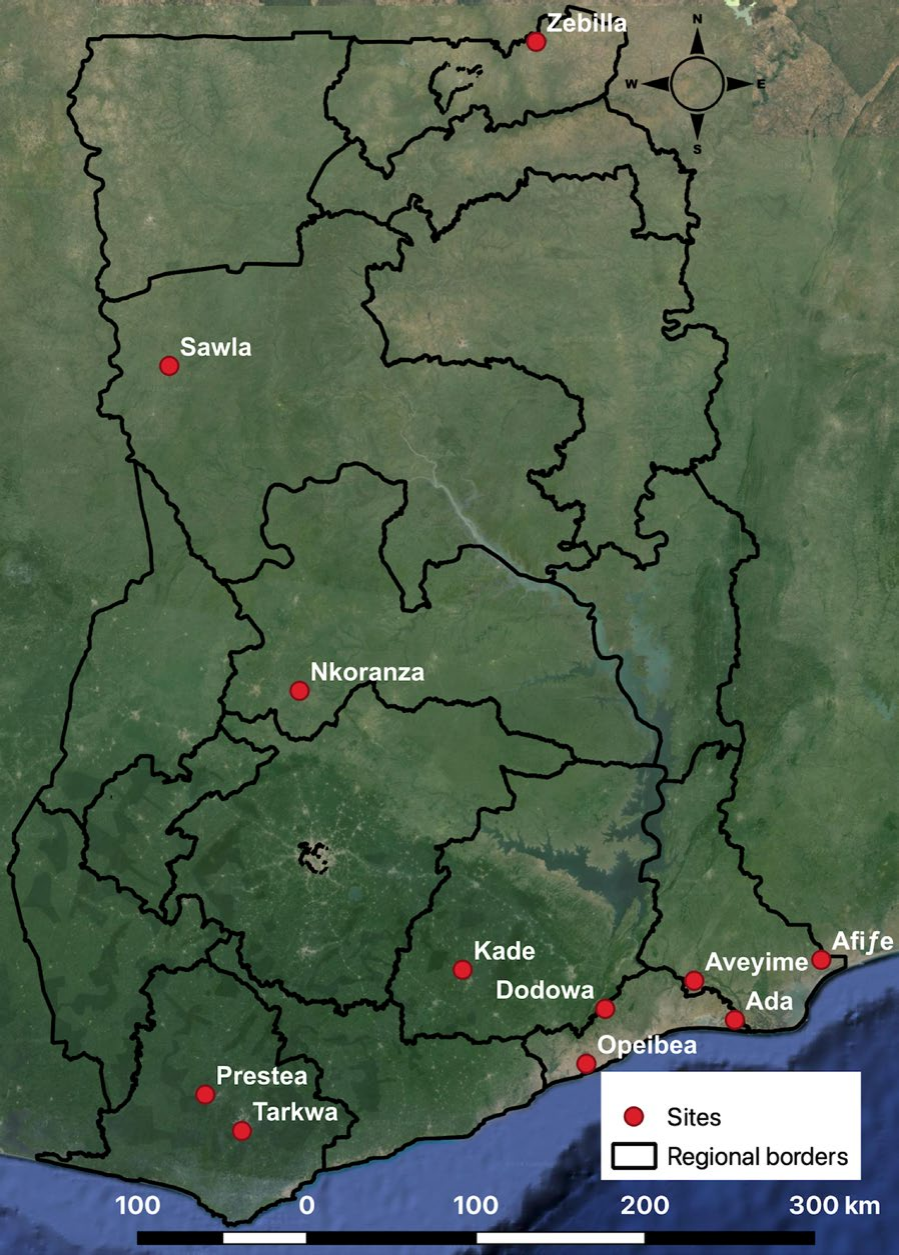
Map of Ghana showing towns where mosquito breeding sites were inspected for larval and pupal collections. The map was generated with ArcGIS software using GPS co-ordinates that were obtained with the KoboCollect app

The study was carried out in 10 areas spread across the six agro-ecological zones of Ghana (Fig. 1): Zebilla (Sudan Savannah), Sawla (Guinea Savannah), Nkoranza (Forest-Savannah Transition zone), Tarkwa and Prestea (Rain Forest), Kade and Aveyime (Semi-deciduous), Ada, Afife and Dodowa (Coastal Savannah). Most of the  sites were selected out of convenience because they were  visited periodically for on-going malaria surveillance activities. A few were also chosen because of previous detection of *Microsporidia MB* in archived mosquito DNA samples [6]. Two rice irrigation sites were purposely included to evaluate whether *Microsporidia MB* infection correlated with these fields, as suggested from the results of a previous study [5].

### Mosquito sampling

*Anopheles* larvae and pupae were collected from breeding sites within the study areas. Sampling was conducted in two consecutive years: August to September 2021 and September to October 2022. These months were within periods following the peak rainfall in Ghana (Additional file 1: Figure S1). The amount of time required per collection period to obtain appreciable numbers of *Anopheles*-positive breeding sites for the study varied across the study areas, and we spent between 2 and 12 days to identify 5 to 13 breeding sites per area per collection year. The GPS co-ordinates of each breeding site was recorded using the KoBoCollect app. *Anopheles* larvae and pupae were collected by the standard dipping method using a 350-mL standard dipper or ladle and then transferred into separate transparent plastic containers with lids that were labeled according to breeding site. In field-improvised insectaries or in the Vector Labs at the Noguchi Memorial Institute for Medical Research (NMIMR), the contents of the plastic containers were transferred into labeled breeder cups and reared to adults. The emerged adult mosquitoes were initially identified based on basic morphology of the leg and wing band patterns using established keys [31]; once identified, they were euthanized by freezing at − 20 °C and stored in 1.5-ml tubes with 70% ethanol. Female and male mosquitoes were separated into different tubes for each breeding site.

### Measuring water physicochemical parameters

At each breeding site, selected physicochemical parameters of the mosquito breeding water were determined prior to collecting immature mosquitoes. Water temperature, pH and dissolved oxygen (DO) level were measured in situ using a thermometer (Fisherbrand traceable Lollipop thermometer, Thermo Fisher Scientific, USA), pH meter (Ohaus pH meter starter pen; OHAUS Corp., Parsippany, NJ, USA) and dissolved oxygen meter (Setsczy), respectively, and recorded. After collecting mosquitoes from a site, we collected 2 L of the mosquito breeding water into plastic bottles and stored the water on ice while in the field. These water samples were refrigerated upon return to the laboratory at NMIMR. The water samples were submitted to the Water Research Institute of the Council for Scientific and Industrial Research, Ghana to measure other water parameters, such as biochemical oxygen demand (BOD), turbidity, salinity, nitrate-nitrogen (NO3-N), phosphate-phosphorus (PO4-P), sulfate (SO4), ammonium-nitrogen (NH4-N) and manganese (Mn), copper (Cu), calcium (Ca) and zinc (Zn) ions. These parameters were selected based on the reported germination requirements of some microsporidians [32–34] and were limited due to the availability of funding.

### DNA extraction from mosquitoes

Adult mosquitoes were retrieved from storage and surface-sterilized in 5% bleach, 70% ethanol and 1× sterile phosphate-buffered saline (PBS). Each mosquito was sectioned into two parts, namely the head + thorax and the abdomen, respectively, with each part placed separately into 1.5-mL tubes. This strategy was adopted to reduce mosquito DNA contamination that would result from using the whole mosquito body because the mosquito abdomen contains the reproductive tissues and midgut where most *Microsporidia MB* are localized [4, 5, 7]. Total DNA was extracted following an adapted non-column protocol using the DNeasy Blood and Tissue DNA reagents (Qiagen, Hilden, Germany). Mosquito abdomens were rinsed briefly in sterile 1× PBS to remove any excess ethanol. The PBS was then discarded, and 1-mm diameter glass beads and 180 µL of ATL lysis buffer were added to the tubes. A blank (tube without a mosquito sample) was included with each set of samples being extracted to check for cross-contamination. The samples were homogenized in a Fisherbrand™ Bead Mill 24 Homogenizer (Thermo Fisher Scientific, Waltham, MA, USA) and then centrifuged at 12,000 *g* for 20 min. After centrifugation, the supernatant of the homogenized samples was carefully transferred into new tubes, 20 µL of proteinase K (10 mg/mL)was added and the tubes were incubated at 56 °C overnight. A 200-µL aliquot of buffer AL was added to each sample, and the samples mixed by vortexing and incubated at 70 °C for 10 min. The samples were then centrifuged at 12,000 *g* for 15 min and supernatants carefully transferred into new tubes. A 300-µL aliquot of ice-cold isopropanol was added to each tube, and the tubes mixed, incubated at − 40 °C for 1 h and then centrifuged at 12,000 *g* for 30 min at 4 °C. The supernatant of each tube was subsequently discarded, and the DNA pellet was washed with washing buffers AW1 and AW2 for 5 min each. The DNA pellet was dried at 50 °C for 2–4 min, reconstituted in 50 µL AE buffer and stored at − 80 °C for future use.

### Identification of mosquito species

Molecular identification of mosquito species was performed with extracted DNA using PCR–restriction fragment length polymorphism (RFLP) [35] followed by short interspersed nuclear elements (SINE) PCR [36] to further distinguish between *An. gambiae* and *Anopheles coluzzii*. PCR products were run in 2% agarose gels stained with SYBR Safe and visualized on an ultraviolet (UV) transilluminator. All samples that could not be identified to species using the methods above were submitted to the ANOSPP [37, 38] platform at the Wellcome Sanger Institute for identification.

### Detection of *Microsporidia MB*

Mosquito DNA was pooled (5–10 mosquitoes) in equal volumes according to breeding site for the detection of *Microsporidia MB*. When a pool tested positive for the presence of the fungus, all samples from that pool were tested individually to enable the frequency of infection to be estimated accurately. Using established *Microsporidia MB*-specific primers [5], *Microsporidia MB* DNA was amplified in a 20-µL reaction volume consisting of 10 µL Luna Universal qPCR Master Mix (NEB, Ipswich, MA, USA; catalog no. M3003), 0.5 µLeach of 10 µM forward and reverse primers, 1 µL of DNA template and 8 µL of nuclease-free water. The real-time PCR (RT-PCR) cycling conditions consisted of an initial denaturation at 95 °C for 5 min, followed by 35 cycles of denaturation at 95 °C for 1 min, annealing at 62 °C for 90 s and extension at 72 °C for 60 s, with a final elongation at 72 °C for 5 min. Melting curves were generated at temperatures ranging from 65 °C to 95 °C. Since RT-PCR was used qualitatively for detection, we used the amplification of the *Anopheles* ribosomal S7 gene with the S7F (5′-TCCTGGAGCTGGAGATGAAC-3′) and S7R (5′-GACGGGTCTGTACCTTCTGG-3′) primers as a DNA quality control. Positive controls used in the reaction were *Microsporidia MB* DNA obtained from Jeremy Herren’s laboratory in KEMRI. Samples were considered to be positive for *Microsporidia MB* if their melting curve matched that of the positive control.

### Data analyses

Data were analyzed using custom scripts in R statistical software [39]. Chi-square tests were used to assess the independence of mosquito proportions to *Microsporidia MB* status, study sites and mosquito sex. Breeding sites from each study area were regarded as replicates. The median proportions of mosquito species identified and *Microsporidia MB* frequencies were compared with the Kruskal–Wallis test. All categorical factors, i.e. site, species and mosquito sex, were fitted into a generalized linear model (GLM) in a binary outcome formula to identify those that significantly correlated with the probability of detecting *Microsporidia MB*. A binomial distribution was fitted using all the explanatory variables singly and then as interaction terms.

Principal component analysis (PCA) biplots were used to show the correlation of water physiochemical parameters with *Microsporidia MB* infection. Multivariate binary logistic regression models were fitted to determine the odds of *Microsporidia MB* positivity given the parameters tested. Four models were fitted and evaluated as follows: model 1 = all water parameters; model 2 = reduced from model 1, using only parameters that showed significance or marginally significant (< 0.1) contribution in model 1 following the analysis of variance (ANOVA) test; models 3 and 4 were expanded forms of model 1 and 2, respectively, where ‘site’ was included to allow downstream analyses of predicted probability of *Microsporidia MB* at each study area. All models were compared with the ‘tab_model’ function in the *sjPlot* R package [40], and the best model was selected based on the Akaike Information Criteria (AIC) instead of the pseudo-*R*^2^ values because of the different number of parameters in each model, and to control for overfitting due to complexity. Receiver operating characteristic (ROC) curves were generated to show the accuracy of the selected models. Predicted probabilities of *Microsporidia MB* infection were estimated for each study site as a function of significant predictors in each selected model.

## Results

### *Anopheles* species distribution

**Fig. 2:**
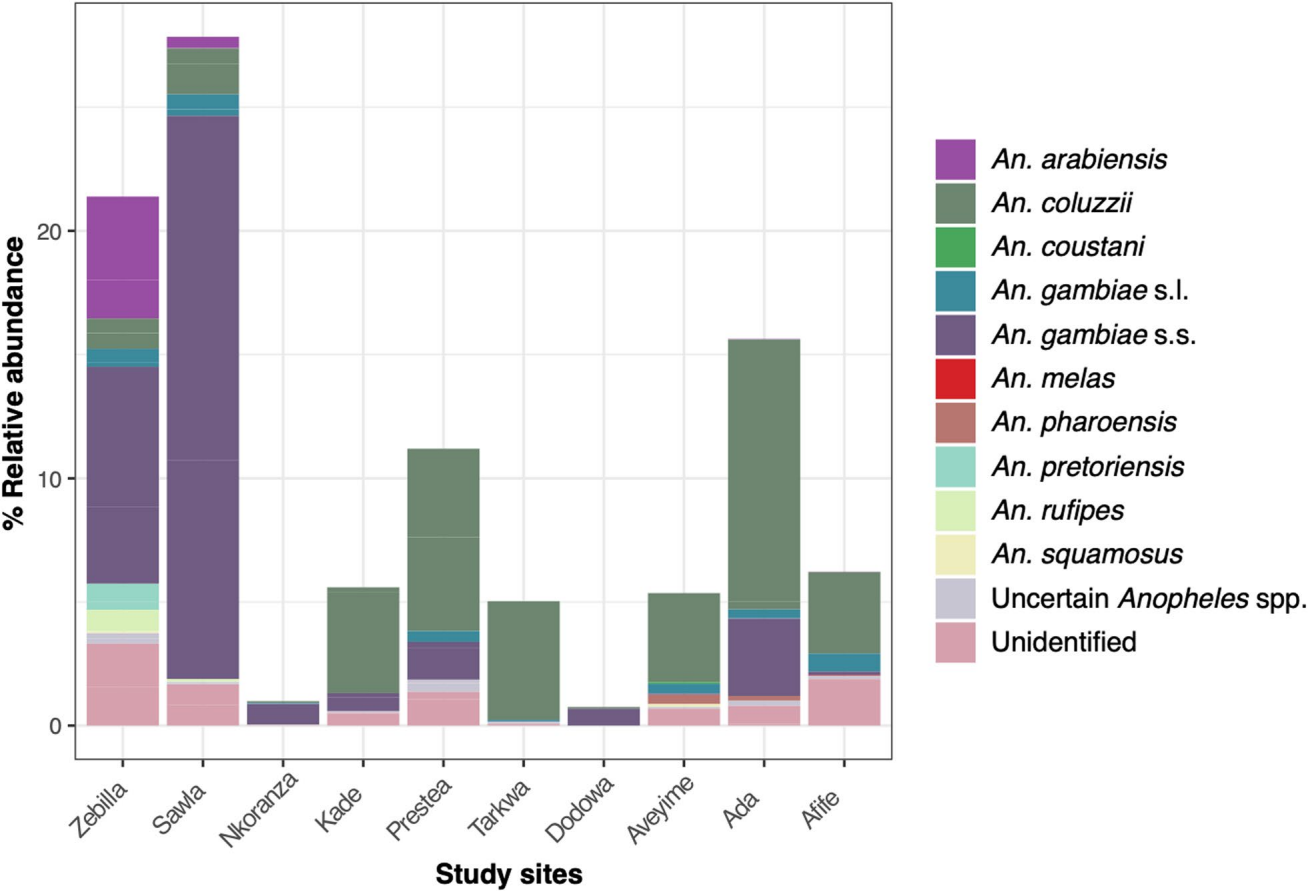
Frequencies of *Anopheles* mosquitoes across study sites. Each bar represents the total percentage of mosquitoes from the collection attributed to a specified site. Stacked bars are colored according to species and show the percentage of *Anopheles* spp. contributed by each site. The study sites are arranged from the northernmost to the southernmost regions (Fig. 1)

A total of 4255 adult mosquitoes were obtained from rearing of the immature stages that were collected from breeding sites (Additional file 2: Table S1). Mosquito counts did not statistically differ between the sampling years (Chi-square test;* χ*^2^ = 20,* df* = 16, *P* = 0.22), but their proportions per site (relative to the total number of mosquitoes collected) were different between study sites (Kruskal test;* χ*^2^ = 66.44,* df* = 9, *P* < 0.0001). Nine confirmed and distinct *Anopheles* species were identified (Fig. 2), which included the minor species *An. coustani*, *An. pharoensis*, *An. pretoriensis*, *An. rufipes* and *An. squamosus*, together accounting for 2.9% of the mosquitoes analyzed. The predominant *An. gambiae* sensu stricto (*An. gambiae* s.s.) (38.6%), *An. coluzzii* (37.4%) and *An. arabiensis* (5.4%) were mostly identified through SINE PCR and RFLP while the other species were distinguished with the ANOSPP algorithm. About 3.7% of the samples were determined to be *An. gambiae* sensu lato (*An. gambiae* s.l.), and 1.6% were identified broadly as belonging to the genus *Anopheles* but could not be further distinguished. About 10% of mosquito samples remained unidentified. Zebilla, which is in the Sudan Savannah eco-zone in the north of the country, had the highest species richness, while Dodowa, in the Coastal Savannah in the south of the country, was the least diverse (Fig. 2). Mosquito species distribution, however, showed no association with ecological zones (Kruskal test;* χ*^2^ = 10.81,* df* = 5, *P* = 0.05).

### *Microsporidia MB* frequency and distribution

**Fig. 3:**
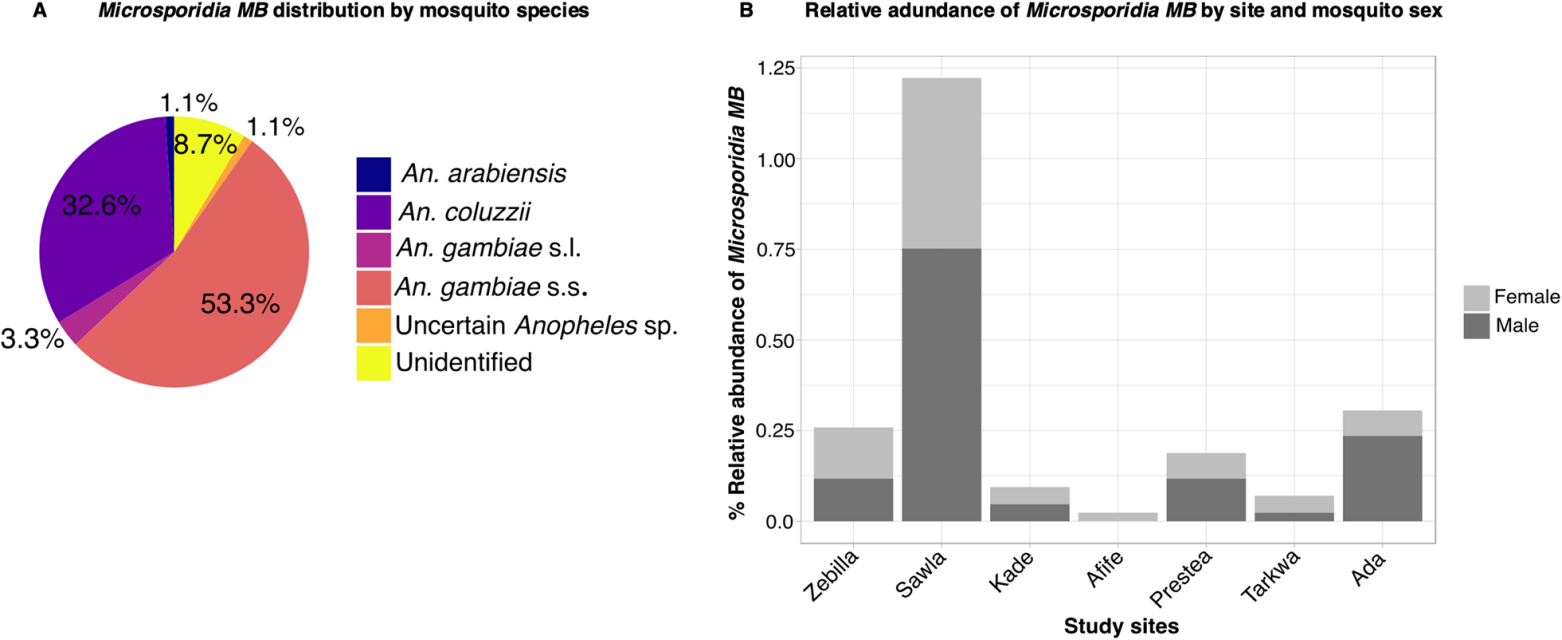
*Microsporidia MB* distribution among mosquito species, study sites and mosquito sex.** A** Pie chart of the percentage of mosquito species found to be infected.** B** Distribution of the total *Microsporidia MB* abundance according to site and mosquito sex

Overall *Microsporidia MB* infection was 2.2% (92/4255) and was detected in six groups of mosquitoes, including the unspecified *Anopheles* species and mosquito samples that were unidentified. Approximately 53% of the infected mosquitoes were *An. gambiae* s.s. and 32% were *An. coluzzii* (Fig. 3a). Only one *An. arabiensis* (1/92) was positive for *Microsporidia MB*, representing 1.1% of the total number of mosquitoes infected with the fungus. Despite this, *Microsporidia MB* positivity did not show significant association with mosquito species (Chi-square;* χ*^2^ = 12.27,* df* = 11, *P* = 0.34).

*Microsporidia MB* positivity was associated with the study site (Chi-square;* χ*^2^ = 43.83,* df* = 9, *P* < 0.0005) and ecological zone (Chi-square;* χ*^2^ = 39.66,* df* = 5, *P* < 0.0005), suggesting that infections were more likely to be found in mosquitoes from some sites than from others. However, the data showed no differences in the observed *Microsporidia MB* frequencies among sites and ecological zones (Kruskal test; *P* > 0.05), implying that higher numbers of mosquitoes are probably required to observe significant differences in *Microsporidia MB* infections between sites. A higher number of males were *Microsporidia MB* positive (59% males vs 40% females;* χ*^2^ = 8.432, *P* = 0.004) (Fig. 3b).

### Prediction of* Microsporidia MB* positivity in study sites, mosquito sex and species

**Fig. 4:**
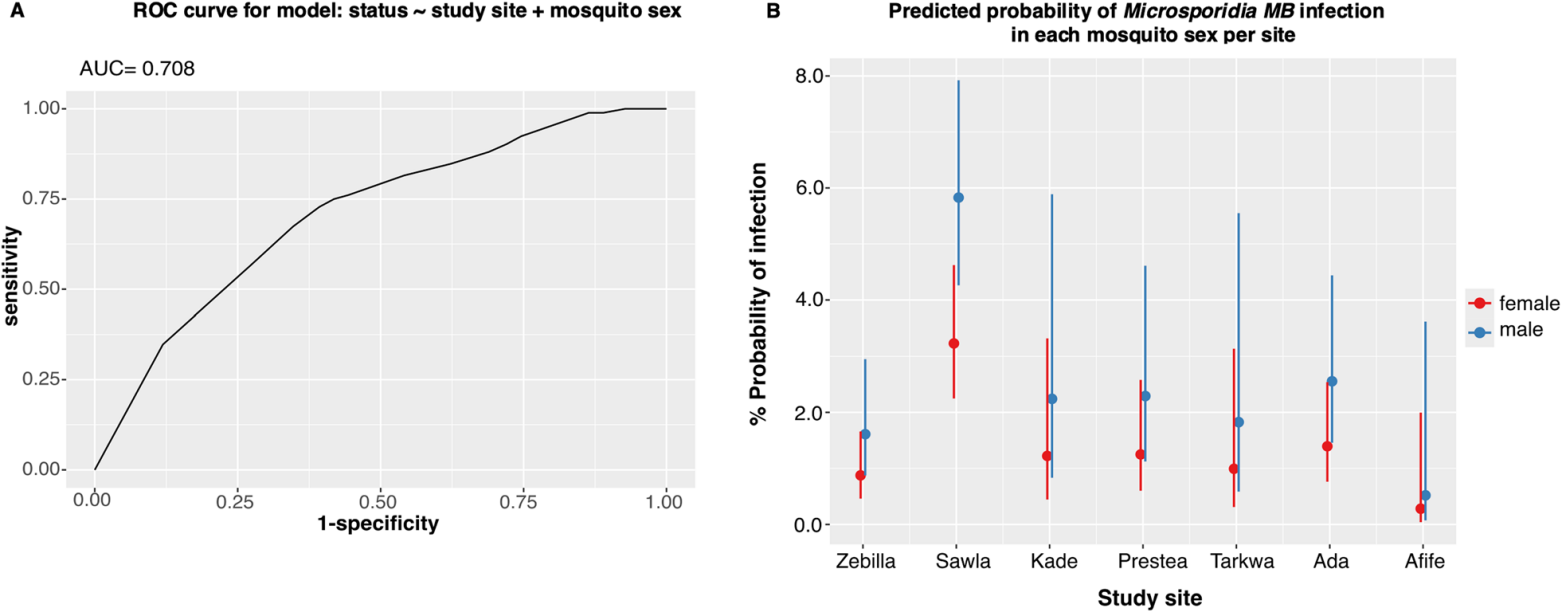
Assessment of the selected model for explaining *Microsporidia MB* status based on study site and mosquito sex.** A** Receiver operating characteristic (ROC) curve showing area under the curve (AUC), which depicts the accuracy of the model.** B **The predicted probabilities of detecting *Microsporidia MB* infection in mosquitoes from sampled sites, showing that the odds for *Microsporidia MB* positivity status are higher in males across all sites

Given the study site, mosquito sex and species, the model that best explained the occurrence of *Microsporidia MB* was the one that included study site and mosquito sex as multivariate non-interaction terms (Additional file 1: Table S2). In this model, study site and mosquito sex, but not mosquito species, were significant factors that correlated with *Microsporidia MB* positivity (Additional file 1: Table S3). Male mosquitoes demonstrated the highest odds ratio (OR) for *Microsporidia MB* infection (OR 1.85, 95% confidence interval [CI] 1.22, 2.85; *P* = 0.004), with a probability that was approximately 85% higher than in females (Table 1). With reference to Zebilla, where 12% of the total *Microsporidia MB* abundance was recorded, Sawla was the only site with significant odds for finding infected mosquitoes [OR 3.78; 95% CI 2.04, 7.68; *P* < 0.001), with 275% higher probability. The model was 71% accurate (Fig. 4a), and predicted that overall, a male mosquito had a 3.23% chance of *Microsporidia MB* infection while a female had 1.61%. Each study site, however, showed varied probabilities, with the highest in Sawla and the probability almost non-existent in Nkoranza, Dodowa and Aveyime (Fig. 4b; Additional file 1: Figure S2).

**Table 1:** Summary characteristics of regression model results for *Microsporidia MB* positivity according to study sites and sex of mosquito

Characteristic	Odds ratio	95% Confidence interval	*P*-value
*Site*			
Zebilla	–	–	
Sawla	3.78	2.04, 7.68	< 0.001
Nkoranza	0	0.00, 5.9E1 + 18	> 0.9
Kade	1.4	0.39, 4.14	0.6
Prestea	1.43	0.55, 3.56	0.4
Tarkwa	1.14	0.26, 3.68	0.8
Dodowa	0	0.00,2.26E + 22	> 0.9
Aveyime	0	0.00, 263	> 0.9
Ada	1.6	0.71, 3.67	0.3
Afife	0.32	0.02, 1.65	0.3
*Sex*			
Female	–	–	
Male	1.85	1.22, 2.85	0.004

### Physicochemical parameters of the mosquito breeding water among study sites

**Fig. 5:**
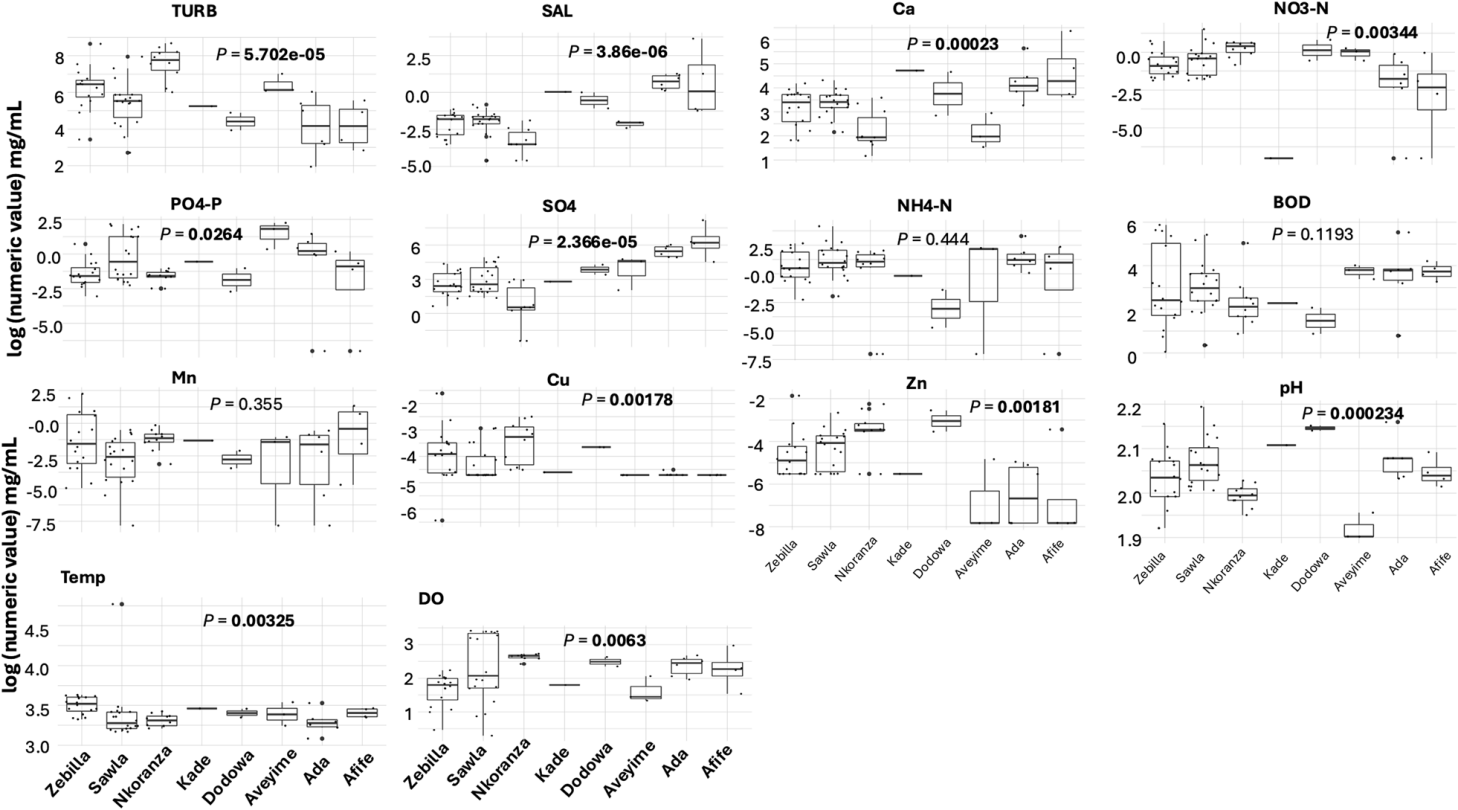
Boxplots of estimated water parameters in the study sites showing the distribution of the physicochemical parameters among breeding sites at each study area. The horizontal line depicts the median value. *P*-values were calculated with the Kruskal-Wallis test to compare the median among study sites for each parameter. Significant *P*-values are shown in bold. BOD, Biochemical oxygen demand; DO, dissolved oxygen; NH4-N, ammonium-nitrogen; NO3-N, nitrate-nitrogen; PO4-P, phosphate-phosphorus; SAL, salinity; SO4, sulfate; TURB, turbidity

A few measured water parameters were below the detectable limits, and to allow plotting and further analyses, such values were replaced with the next smallest positive number (Additional file 3: Table S4). All the parameters showed significant differences among the study sites except, biochemical oxygen demand (BOD), ammonium-nitrogen (NH4-N) and manganese (Mn) (Fig. 5). Nkoranza differed most with Ada and Afife, being only similar in the PO4-P, DO concentrations and temperature (Additional file 4: Table S5). Salinity was the most variable parameter, followed by sulfate (SO4), with the latter being generally higher as one moved from the north towards the south (Fig. 5).

### Correlation of* Microsporidia MB* positivity with physicochemical parameters of the mosquito breeding water

**Fig. 6:**
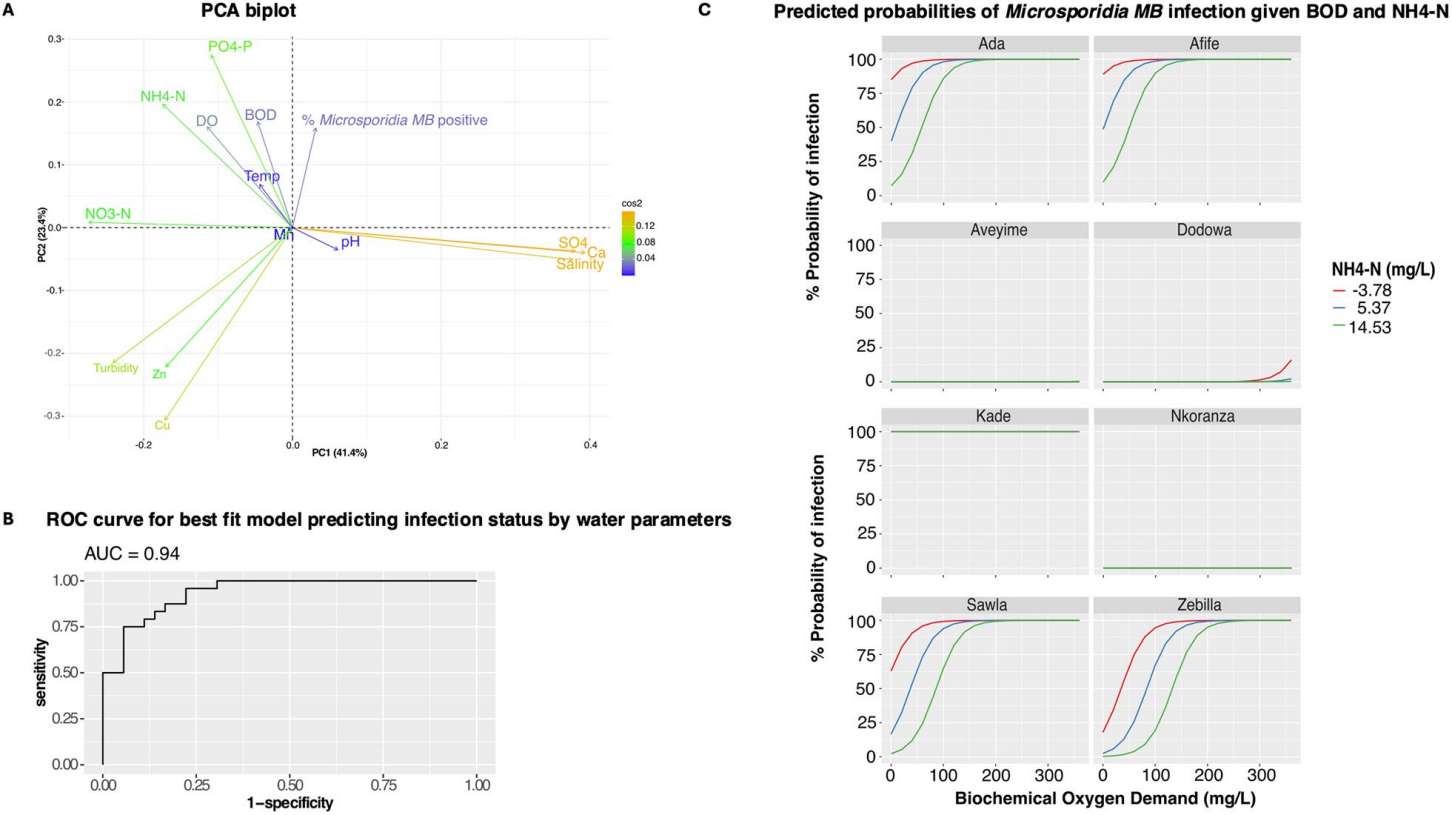
Results from the best selected model that explains probability of detecting *Microsporidia MB*-infected mosquitoes based on physicochemical parameters of the mosquito breeding water.** A** Principal coordinates analysis (PCA) biplot of the correlation between *Microsporidia MB* and the quantitative parameters. Arrows show the direction of each variable in relation to the principal components (PC), and the lengths of the arrows represent the strength of the contribution of the variable to the PC. Arrows close together are positively correlated with each other, while those opposite each other are negatively correlated. Variables at a 90º angle have no correlation.** B** Receiver Operating Characteristic (ROC) curve shows the area under the curve (AUC).** C** Predicted probabilities of detecting *Microsporidia MB* infection in mosquitoes from sampled sites based on arbitrary BOD and NH4-N. BOD, Biochemical oxygen demand; DO, dissolved oxygen; NH4-N, ammonium-nitrogen; NO3-N, nitrate-nitrogen; PO4-P, phosphate-phosphorus; SO4, sulfate

We visualized the correlation between the water parameters and the proportion of *Microsporidia MB*-infected mosquitoes using PCA biplots. The first two axes explained approximately 65% of the total variance observed in the data, with Ca ions, SO4 and salinity contributing most to the first principal component (Fig. 6a). BOD, PO4-P, NH4-N, DO, Mn and temperature were positively correlated with *Microsporidia MB*. The remaining parameters tested, with the exception of pH, SO4, Ca ions and salinity, had a negative relationship with the presence of the fungus (Fig. 6A).

Based on the best model (Additional file 1: Table S6) which incorporates turbidity, PO4-P, SO4, NH4-N, BOD, Mn, Cu, Zn, DO and site as explanatory variables for *Microsporidia MB* status, the odds for detecting *Microsporidia MB* increases by a factor of 1.04 with every unit increase in BOD [OR (95% CI) = 1.04 (1.02, 1.10); *P* = 0.02] and decreases with increasing units of NH4-N (Table 2). With 94% accuracy (Fig. 6B), the model predicts varied probabilities for *Microsporidia MB* infection at the sampled sites which appears dependent on a site-related interaction between the BOD and NH4-N (Fig. 6c; Additional file 4: Table S5).

**Table 2:** Summary characteristics of regression model results for *Microsporidia MB* positivity explained by a number of the breeding water parameters

Characteristic	Odds ratio	95% Confidence interval	*P*-value
TURB	1.00	1.00, 1.00	0.4
PO4-P	1.43	0.59, 9.03	0.5
SO4	0.99	0.98, 1.00	0.2
NH4-N	0.79	0.57, 0.96	0.048*
BOD	1.04	1.02, 1.10	0.016*
Mn ion	0.33	0.05, 0.81	0.083
Cu ion	1.77E + 04	0.00, 3.36E + 31	0.8
Zn ion	0.00	0.00, 1.37E + 06	0.2
DO	1.16	0.93, 1.53	0.2
*Site*			
Ada	—	—	
Afife	1.42	0.02, 95.1	0.9
Aveyime	0.00		> 0.9
Dodowa	0.00		> 0.9
Kade	4.91E + 07	0.00, NA	> 0.9
Nkoranza	0.00		> 0.9
Sawla	0.30	0.00, 23.1	0.6
Zebilla	0.04	0.00, 2.94	0.2

*BOD* Biochemical oxygen demand,* DO* dissolved oxygen,* NA* not available, * NH4-N* ammonium-nitrogen,* PO4-P* phosphate-phosphorus,* SO4* sulfate,* TURB* turbidity

*Significant at* P* ≤ 0.05

## Discussion

*Microsporidia MB* infection among mosquitoes is reported to be generally low [5–7] with seasonal variations that shows increased distribution among mosquitoes after rainfall [5]. As rainfall also influences the range and dynamics of mosquito breeding sites, identifying micro-environmental factors in mosquito breeding water that impact the sustainability of *Microsporidia MB* among mosquitoes could contribute to a clearer understanding of how this micro-organism can be used effectively in mosquito-borne disease control. Our data shows that *Microsporidia MB* positivity is not a random occurrence, but it is associated with the physicochemical properties of mosquito breeding sites. Among the parameters tested, BOD was found to have a significant positive relationship with *Microsporidia MB* and, depending on the breeding site, Mn ion and ammonium-nitrogen NH4-N could also be marginally inversely involved. In agreement with previous reports, we also found male mosquitoes to have a higher probability of *Microsporidia MB* infection than females irrespective of the study area, independent of the *Anopheles* species.

Microsporidians are largely obligate intracellular parasites, which implies that they need a host cell to survive. Their spores are environmentally resistant and metabolically inactive until germination and are the only microsporidian life stage that has been found to be viable outside of a host cell [41]. Microsporidians, therefore, require hosts to be in their optimal growth environment to ensure their sustainability, without necessarily using the resources available in the host’s surroundings [34]. In the present study, we found that BOD significantly raised the odds of detecting *Microsporidia MB-*infected larvae. BOD is a measure of the amount of oxygen available to micro-organisms during the decomposition of organic matter; as such, BOD is an important parameter for assessing organic pollution in water. While high BOD can harm aquatic life due to a reduced DO level, BOD has also been found to be linked to *Anopheles* larvae abundance [27, 42]. High organic content is essential for larval nutrition and protects larvae against UV radiation [43]. Together, these findings suggest that there is an increased chance of detecting *Microsporidia MB*-infected larvae in environments that are very conducive for *Anopheles* breeding. Such sites are likely to be those that are densely populated with *Anopheles* larvae. Given the generally low distribution of infection in Ghana [6], this implication is not surprising. Relatively more mosquitoes need to be collected to detect *Microsporidia MB* infection, which is more probable where large numbers of mosquitoes are found. BOD levels above 305 mg/l are, however, detrimental to *Anopheles* larvae [44], and such sites are not preferred by ovipositing *Anopheles* female mosquitoes in nature [45]. The BOD levels measured in the present study were generally well below this experimental value, with an average BOD level that was more than twofold higher at *Microsporidia MB*-positive sites (79.4 mg/l) than at *Microsporidia MB*-negative sites (32.8 mg/l). It will be useful to titrate the limits of BOD for mosquito abundance and *Microsporidia MB* detection to better inform its use for surveillance and evaluation of control activities.

According to our regression model, NH4-N showed decreased odds for *Microsporidia MB*-infected mosquitoes. Ammonium (NH4) gets into the mosquito aquatic environment through both anthropological and mosquito physiological processes. Mosquito larvae excrete ammonium into their breeding water; consequently, overcrowding would lead to increased NH4 ion concentrations to levels that become toxic to the larval population [46, 47]. Survival of larvae also decreases significantly with extraneous addition of high concentrations of NH4 to the breeding water [48] such as, for example, through agricultural fertilizers [49]. This could partly explain why some study sites, particularly those where agricultural activities were observed, such as in Aveyime and Afife, would have low mosquito numbers. However, these sites were sampled in only one collection period, which limited the total number of mosquitoes obtained in the study. Both NH4 and SO4 have been shown to correlate with increased abundance of *An. arabiensis* and *Culex* spp. in rice fields [49] and nitrogen has been shown to correlate with *Culex* sp. in agricultural wastewater [50]. Our results could not corroborate this association because *An. arabiensis* accounted for a low proportion of our catch, and those that were found were mostly collected at non-rice field sites. If the estimated water parameters are associated with *Microsporidia MB* as a direct function of mosquito abundance, then *Microsporidia MB* frequency would be expected to increase with increasing NH4-N and SO4 levels. In addition to increased levels of NH4, high SO4 also prevents larval development of *Anopheles* mosquitoes [45, 51]; in the present study, this was demonstrated by a negative correlation between *Microsporidia MB* status and SO4. In fact, SO4 estimates were on average approximately 1253% higher in rice fields (694.7 mg/l) than in non-agricultural sites and approximately 96% higher in *Microsporidia MB*-negative sites (156.7 mg/). *Microsporidia MB* positivity also correlated positively with NH4-N, but the probability of *Microsporidia MB* decreased with increasing levels of this ion, indicating that NH4-N could be a good indicator for mosquito abundance and the presence of *Microsporidia MB*. The levels of NH4-N also need to be clearly defined to identify the range of concentrations that are conducive for sustaining *Microsporidia MB*. According to our physicochemical parameter model, it appears that with similar BOD and NH4-N across sites, there are other ecological factors that may not have been evaluated in this study that would explain the varied probabilities of finding *Microsporidia MB*-infected mosquitoes at these sites. Extensive studies at agricultural sites could provide more insight into their association with the abundance of dominant *Anopheles* species, such as *An. gambiae*, *An. coluzzii* and *An. funestus*, and *Microsporidia MB* infectivity. Such studies are needed as the results of the present study are contrary to what has previously been shown for *An. arabiensis* in Kenya [5]; NH4, BOD and SO4 concentrations at these sites could also have negative implications on the use of the fungal symbiont to disease control.

*Microsporidia MB* has so far been isolated only from *Anopheles* mosquito species and, in particular, in dominant malaria-transmitting vectors, including *An. funestus* [4–6]. *Plasmodium* infections have also been detected in other members of the *Anopheles gambiae* complex, such as *An. pharoensis*, *An. rufipes*, *An. squamosus* and *An. coustani*, all of which are mostly exophilic [52–55]. While the contribution of these latter mosquito species to the epidemiology of malaria in a geographical area may be negligible in most malaria endemic regions due to their relatively low numbers, these minor vectors are also affected by control activities that are primarily designed to target the major vectors. The wide range of mosquitoes identified with the improved ANOSPP algorithm has allowed us to show, for the first time, that these minor species are equally likely to be infected with *Microsporidia MB*. Our results suggest that the species of mosquito is not a significant determining factor for *Microsporidia MB* positivity and that the probability of *Microsporidia MB* positivity is simply higher in any *Anopheles* male mosquito. A fruitful line of research would be to investigate the frequency of *Microsporidia MB* infections in geographical locations where these vectors are found in high numbers to confirm that these vectors can also be infected with the fungus. The results of such studies will further expand the range at which *Microsporidia MB*-*Anopheles* research can be extended for a more inclusive *Microsporidia MB*-mediated control strategy [3].

## Conclusions

The implications for mosquito populations naturally infected with *Microsporidia MB* are highly promising for future control mechanisms involving this endosymbiont. Our study used regression models to show that a number of biotic and abiotic factors can be incorporated into surveillance models for mosquito larval source management, thus maximizing the detection of *Microsporidia MB*-infected populations in a geographical location. Such models will also be useful in assessing the feasibility and field dynamics of using *Microsporidia MB* for control, especially with increasing chemical use in agricultural activities [56].

## Supplementary Information

**Additional file 1: Figure S1: **Monthly average rainfall information for Ghana in 2021 (blue) and 2022 (green) obtained from ClimateEngine.org (https://www.climateengine.org/).** Figure S2: **Predicted probabilities of *Microsporidia MB* infection in study sites according to the selected model status ~ site + sex.  **Table S2:** Comparison of logistic regression models fitted to identify significant categorical factors (study site, mosquito species and sex) correlating with *Microsporidia MB* positivity. **Table S3:** Results for test of statistical relationship between study site, mosquito species and sex as interaction terms for *Microsporidia MB* occurrence. **Table S6:** Comparison of outputs for models on water abiotic parameters association with *Microsporidia MB*.

**Additional file 2: Table S1:** Summary of mosquito collected and *Microsporidia MB* relative abundance per site.

**Additional file 3: Table S4:** Estimated water parameters for selected breeding sites at the study areas.

**Additional file 4: Table S5:** Results of Dunn test with Benjamini-Hochberg correction for pairwise comparisons, following Kruskal Wallis test of variance for water parameters among the study sites.

## Data Availability

The dataset(s) supporting the conclusions of this article is(are) included within the article (and its additional file(s)).
