# Supplementary material for: Mosquito breeding water parameters are important determinants for Microsporidia MB in the aquatic stages of Anopheles species
Source: Parasit Vectors. 2024 Dec 18;17:509. doi: 10.1186/s13071-024-06596-9 (PMC11657153; doi:10.1186/s13071-024-06596-9)
Supplement: Supplementary file 1 — Additional file 1: Figure S1: Monthly average rainfall information for Ghana in 2021 (blue) and 2022 (green) obtained from ClimateEngine.org (https://www.climateengine.org/). Figure S2: Predicted probabilities of Microsporidia MB infection in study sites according to the selected model status ~ site + sex. Table S2: Comparison of logistic regression models fitted to identify significant categorical factors (study site, mosquito species and sex) correlating with Microsporidia MB positivity. Table S3: Results for test of statistical relationship between study site, mosquito species and sex as interaction terms for Microsporidia MB occurrence. Table S6: Comparison of outputs for models on water abiotic parameters association with Microsporidia MB. [file 13071_2024_6596_MOESM1_ESM.docx]

**Additional file 1**

**Breeding water parameters are important determinants for *Microsporidia MB* in the aquatic stages of *Anopheles* mosquitoes**

Esinam A. Akorli ^1$^, Nana Efua Andoh ^1,2 $^, Richardson K. Egyirifa¹ Christopher Dorcoo ^1^, Sampson Otoo ^1^, Seraphim N.A. Tetteh ^1^, Reuben Mwimson Pul ^1^, Derrick B. Sackitey ^1^, Stephen K.D Oware ^1^, Samuel K. Dadzie ^1^, Jewelna Akorli ^1^*.

^1^ Department of Parasitology, Noguchi Memorial Institute for Medical Research, University of Ghana, P.O. Box LG 581, Legon Accra

^2^ Department of Pathology, University of Cambridge, 10 Tennis Ct Rd, Cambridge CB2 1QP, United Kingdom

***** Corresponding author: [jakorli@noguchi.ug.edu.gh](mailto:jakorli@noguchi.ug.edu.gh)

$ These authors worked equally and are shared first authors.

**Figure S1:** **Monthly average rainfall information for Ghana in 2021 (blue) and 2022 (green) obtained from ClimateEngine.org (**[**https://www.climateengine.org/**](https://www.climateengine.org/)**).** Red horizonal lines are the annual average precipitation recorded for each sampling year.

2021

2022

2022

2021

**Figure S2: Predicted probabilities of *Microsporidia MB* infection in study sites according to the selected model status ~ site + sex.** All study sites are shown in A while only sites with finite confidence intervals (CI) are shown in B. Red dots represent the odds ratio and, extended vertical red lines are the 95% CI. All sites except Sawla contain CI of 1.

**B**

**A**

**Table S2:** **Comparison of logistic regression models fitted to identify significant categorical factors (study site, mosquito species and sex) correlating with *Microsporidia MB* positivity.** Presence of infection (status) was modelled against site (status ~ site) and sex (status ~ sex) separately, and then as interaction variables (status ~ site + sex).

|  | **status ~ site** | | **status ~ sex** | | **status ~ site + sex** | |
| --- | --- | --- | --- | --- | --- | --- |
| *Predictors* | *Odds Ratios* | *P* | *Odds Ratios* | *P* | *Odds Ratios* | *P* |
| (Intercept) | 0.01 ^***^ | **<0.001** | 0.02 ^***^ | **<0.001** | 0.01 ^***^ | **<0.001** |
| site [Sawla] | 3.75 ^***^ | **<0.001** |  |  | 3.78 ^***^ | **<0.001** |
| site [Nkoranza] | 0.00 | 0.993 |  |  | 0.00 | 0.993 |
| site [Kade] | 1.40 | 0.570 |  |  | 1.40 | 0.567 |
| site [Prestea] | 1.40 | 0.475 |  |  | 1.43 | 0.443 |
| site [Tarkwa] | 1.16 | 0.819 |  |  | 1.14 | 0.846 |
| site [Dodowa] | 0.00 | 0.994 |  |  | 0.00 | 0.994 |
| site [Aveyime] | 0.00 | 0.983 |  |  | 0.00 | 0.983 |
| site [Ada] | 1.63 | 0.237 |  |  | 1.60 | 0.255 |
| site [Afife] | 0.31 | 0.263 |  |  | 0.32 | 0.275 |
| sex [male] |  |  | 1.85 ^**^ | **0.004** | 1.85 ^**^ | **0.004** |
| Observations | 4255 | | 4255 | | 4255 | |
| R^2^ Tjur | 0.010 | | 0.002 | | 0.013 | |
| AIC | 860.451 | | 883.077 | | 854.104 | |
|  | | | | | | |

**Table S3: Results for test of statistical relationship between study site, mosquito species and sex as interaction terms for *Microsporidia* *MB* occurrence.**

| Call: anova(modelst_sp_sx, test="Chisq")  Model: binomial, link: logit  Response: status  Terms added sequentially (first to last) | | | | | | |
| --- | --- | --- | --- | --- | --- | --- |
|  | **Df** | **Deviance** | **Residual Df** | **Residual Deviance** | ***P*-value** |  |
| NULL | 4255 | 5898.70 |  |  |  |  |
| site | 10 | 5058.20 | 4245 | 840.5 | < 2.20e-16 | *** |
| species | 11 | 8.90 | 4234 | 831.6 | 0.635216 |  |
| sex | 1 | 9.90 | 4233 | 821.7 | 0.001695 | ** |

**Table S6:** **Comparison of outputs for models on water abiotic parameters association with *Microsporidia MB*.** Model 1 included all physicochemical variables. Model 2 is reduced from Model 1 and contains variables that showed significant odds ratio following statistical analyses of Model 1. Models 3 and 4 are Models 1 and 2, respectively with Site included as an explanatory variable.

|  | **Model 1** | | **Model 2** | | **Model 3** | | **Model 4** | |
| --- | --- | --- | --- | --- | --- | --- | --- | --- |
| *Predictors* | *Odds Ratios* | *p* | *Odds Ratios* | *p* | *Odds Ratios* | *p* | *Odds Ratios* | *p* |
| (Intercept) | 0.00 | 0.377 | 0.83 | 0.843 | 2285.39 | 0.633 | 2.66 | 0.667 |
| TURB | 1.00 | 0.337 | 1.00 | 0.071 | 1.00 | 0.192 | 1.00 | 0.429 |
| SAL | 0.75 | 0.718 |  |  | 0.43 | 0.596 |  |  |
| Ca | 1.03 | 0.117 |  |  | 1.02 | 0.296 |  |  |
| NO3 N | 0.41 | 0.334 |  |  | 28.30 | 0.249 |  |  |
| PO4 P | 1.01 | 0.956 | 0.93 | 0.711 | 23.23 | 0.419 | 1.43 | 0.525 |
| SO4 | 0.99 | 0.114 | 1.00 | 0.160 | 0.99 | 0.258 | 0.99 | 0.183 |
| NH4 N | 0.99 | 0.923 | 0.95 | 0.448 | 0.40 | 0.197 | 0.79 ^*^ | **0.048** |
| BOD | 1.04 ^*^ | **0.049** | 1.03 ^*^ | **0.029** | 1.08 | 0.052 | 1.04 ^*^ | **0.016** |
| Mn | 0.32 ^*^ | **0.046** | 0.45 | 0.074 | 0.28 | 0.119 | 0.33 | 0.083 |
| Cu | 4.72 | 0.956 | 0.00 | 0.634 | 395.64 | 0.890 | 17661.52 | 0.769 |
| Zn | 0.00 | 0.085 | 0.00 | 0.081 | 0.00 | 0.092 | 0.00 | 0.180 |
| pH | 1.77 | 0.540 |  |  | 0.03 | 0.192 |  |  |
| Temp | 1.03 | 0.478 |  |  | 2.02 | 0.131 |  |  |
| DO | 1.15 | 0.076 | 1.15 ^*^ | **0.043** | 1.05 | 0.840 | 1.16 | 0.231 |
| Site [Afife] |  |  |  |  | 1.59 | 0.915 | 1.42 | 0.858 |
| Site [Aveyime] |  |  |  |  | 0.00 | 0.993 | 0.00 | 0.997 |
| Site [Dodowa] |  |  |  |  | 0.00 | 0.998 | 0.00 | 0.998 |
| Site [Kade] |  |  |  |  | 5607147.73 | 0.999 | 49075008.87 | 0.999 |
| Site [Nkoranza] |  |  |  |  | 0.00 | 0.989 | 0.00 | 0.991 |
| Site [Sawla] |  |  |  |  | 0.89 | 0.969 | 0.30 | 0.610 |
| Site [Zebilla] |  |  |  |  | 0.00 | 0.118 | 0.04 | 0.189 |
| Observations | 60 | | 60 | | 60 | | 60 | |
| R^2^ Tjur | 0.476 | | 0.396 | | 0.685 | | 0.577 | |
| AIC | 75.498 | | 71.997 | | 71.357 | | 70.474 | |
| ** p<0.05   ** p<0.01   *** p<0.001* | | | | | | | | |

*Model 1= glm(status ~ TURB+SAL+Ca.+NO3.N+PO4.P+SO4+NH4.N+BOD+Mn+Cu+Zn+pH+Temp+DO,*

*data = data, family = "binomial"(link = "logit"))*

*Model 2= glm(status ~ TURB+PO4.P+SO4+NH4.N+BOD+Mn+Cu+Zn+DO,*

*data = data, family = "binomial"(link = "logit"))*

*Model 3= glm(status ~ TURB+SAL+Ca.+NO3.N+PO4.P+SO4+NH4..N+BOD+Mn+Cu+Zn+pH+Temp+DO+Site,*

*data = data, family = "binomial"(link = "logit"))*

*Model 4= glm(status ~ TURB+PO4.P+SO4+NH4..N+BOD+Mn+Cu+Zn+DO+Site,*

*data = data, family = "binomial"(link = "logit"))*
